# Evolution of extracorporeal membrane oxygenation: historical milestones and advanced developments

**DOI:** 10.1051/ject/2025033

**Published:** 2025-12-17

**Authors:** Lajos Szentgyorgyi, Salman Pervaiz Butt, Bhuvaneswari Krishnamoorthy

**Affiliations:** 1 School of Health and Society, University of Salford Mary Seacole Building, Frederick Road Campus, Broad St Salford M6 6PU United Kingdom; 2 Manchester University NHS Foundation Trust, Wythenshawe Hospital Southmoor Road Manchester M23 9LT United Kingdom; 3 Heart Vascular and Thoracic Institute, Cleveland Clinic Abu Dhabi Abu Dhabi PO Box 112412 United Arab Emirates; 4 Division of Cardiovascular Sciences, University of Manchester 46 Grafton Street Manchester M13 9NT United Kingdom

**Keywords:** ECMO (extracorporeal membrane oxygenation), History, Cardiopulmonary bypass (CPB), Oxygenator, Perfusion

## Abstract

Extracorporeal membrane oxygenation (ECMO) has a history that is a testament to the pioneering spirit of medical innovators. It is intricately linked with the development of cardiopulmonary bypass (CPB) technology. The journey of ECMO can be traced back to the mid-20th century when experiments with CPB began to support patients undergoing cardiac surgery. However, it was not until the 1970s that ECMO emerged as a standalone therapy. Throughout the following decades, ECMO technology advanced rapidly, with improvements in circuit design, oxygenators, and pump technology enhancing its safety and efficacy. ECMO’s versatility soon became apparent as it was employed in various clinical scenarios, including acute respiratory distress syndrome (ARDS), cardiac failure, and even as a bridge to lung or heart transplantation. In recent years, efforts have focused on miniaturisation, cost reduction, and the development of portable systems, enabling their use outside traditional intensive care settings. Today, ECMO remains not just a tool but a lifeline in the management of life-threatening cardiorespiratory failure. It offers hope and a second chance to patients when conventional therapies fall short, underscoring its critical importance in critical care medicine, cardiology, transplant and cardiothoracic surgery. This article provides a concise yet comprehensive overview of the history and recent advancements in ECMO.

## Introduction

Extracorporeal Membrane Oxygenation (ECMO) is a rapidly evolving technology providing mechanical circulatory and respiratory support for patients with severe respiratory and cardiac failure refractory to conventional management. However, ECMO does not treat the underlying pathology; therefore, it is not considered a treatment. Since its first successful use in 1971 [1], ECMO has revolutionised care for patients with end-stage cardiac and respiratory failures, often requiring transplants [2, 3]. It has also enabled complex surgeries, including tracheobronchial repairs [4–6].

Modern ECMO is a highly advanced form of Extracorporeal Life Support (ECLS) that has evolved over 200 years. It was developed through animal experiments with primitive extracorporeal support and improvements in Cardiopulmonary Bypass (CPB) technology employed during heart surgeries. Like other extracorporeal life support devices, such as CPB machines, haemodialysis, haemofiltration, haemo-adsorption, extracorporeal carbon dioxide (CO_2_) removal (ECCO_2_R), and cardiac ventricular assist devices, ECMO removes and returns blood to the body. However, this review deliberately focuses exclusively on ECMO rather than the broader spectrum of organ-support modalities. Although all these technologies share a common foundation and have progressed via the dedication and ingenuity of pioneering individuals and teams, our focus remains on ECMO’s history, current developments, and future directions. Over time, pioneers have tirelessly worked to refine and optimise extracorporeal membrane oxygenation, providing life-saving interventions for critically ill patients. This article pays tribute to their enduring legacy.

## Unveiling the fundamentals in the 19th century: from experiments to the first closed-system organ perfusion

**Figure 1:**
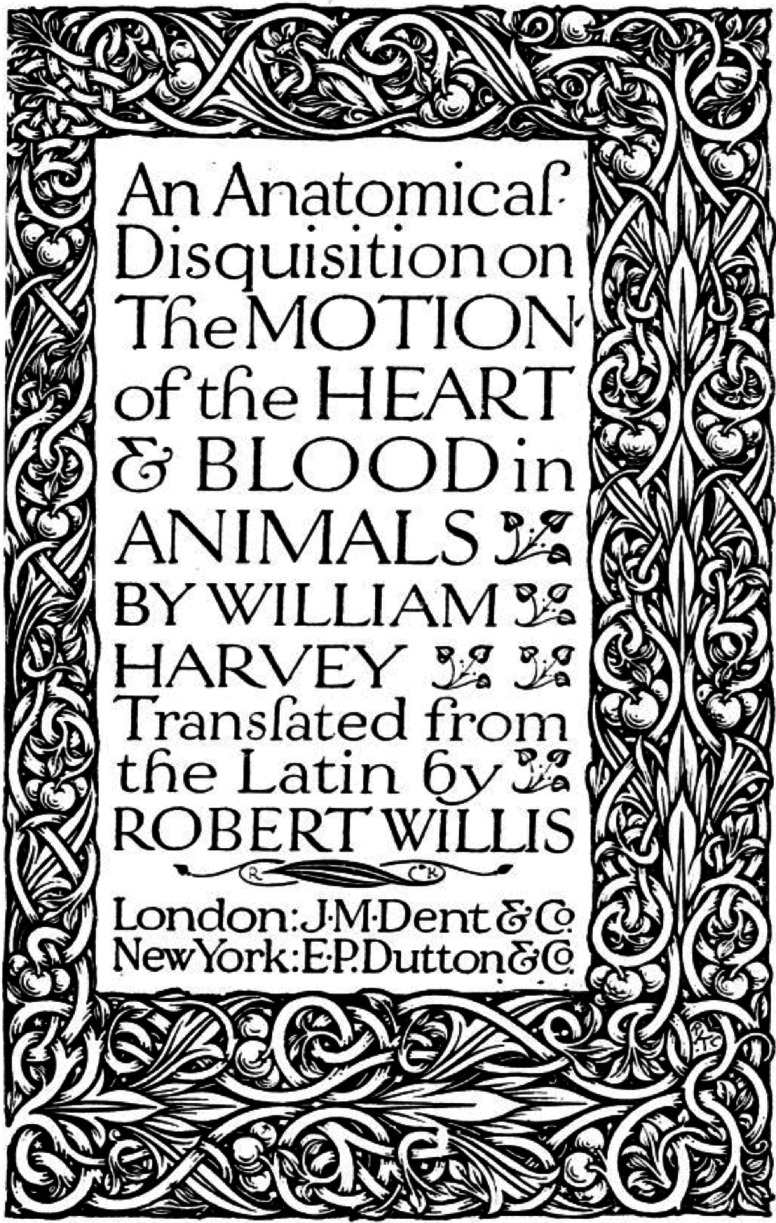
William Harvey: An Anatomical Disquisition On The Motion Of The Heart And Blood In Animals. Book title page. (Available from the public domain in the USA. https://www.gutenberg.org/cache/epub/67065/pg67065-images.html.)

The history of ECLS dates back to significant milestones in medical science. The foundation was laid with William Harvey’s description of the circulatory system in 1628 (William Harvey: An Anatomical Disquisition On The Motion Of The Heart And Blood In Animals; Figure 1) [7]. Julien Jean Cesar Legallois developed the first isolated heart-lung preparation in rabbits in France in 1812. His visionary idea established the basic principle of cardiopulmonary bypass machines. He stated that “*if the place of the heart could be supplied by injection, and if, for the regular continuance of this injection, there could be furnished a quantity of arterial blood, whether natural, or artificially formed… then life might be indefinitely maintained*” [8]. In 1849, Carl Eduard Loebell conducted experiments on isolated pig kidneys, introducing continuous organ perfusions [9]. He used defibrinated blood to perfuse the kidneys. He observed that bright red arterial blood extravasated from the renal veins with a dark colour and higher viscosity, while a clear fluid came out of the ureter. In 1868, Alexander Schmidt at the Physiological Institute in Leipzig oxygenated venous blood by adding oxygen and developed methods to estimate oxygen and carbon dioxide content [9]. The concept of oxygenating blood through contact with air currents led to the creation of a primitive form of bubble oxygenator by Waldemar von Schröder in Strasbourg in 1882. However, he also noted the problems associated with this method: “*Because of the rapid air current passing through the blood, foaming often occurs quite heavily*” [9].

**Figure 2:**
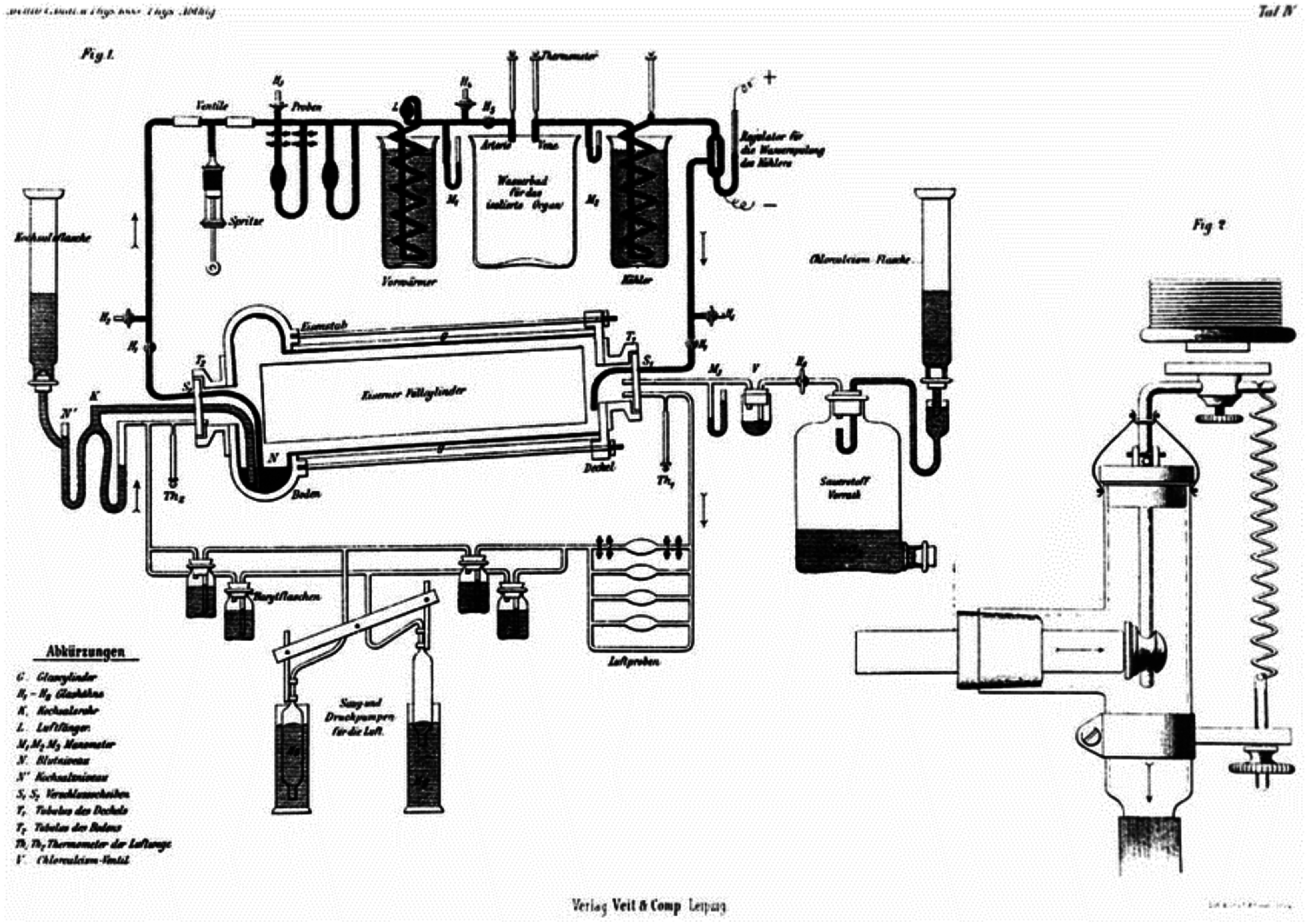
The respiration apparatus of von Frey and Gruber was the first device for organ perfusion. A closed-circuit system features a film oxygenator that receives oxygen via a valve and a gas pressure measuring device. A motor-driven syringe and valves facilitate the continuous circulation of warmed blood before it passes through the isolated organ. An air trap is in place to protect the organ from gas embolism. (Adapted from Boettcher et al., 2003 [9]).

In 1885, Max von Frey and Max Gruber developed the first closed artificial circulation system with a film oxygenator at the Physiological Institute in Leipzig, representing a rudimentary prototype of a heart-lung bypass apparatus (Figure 2) [9]. While it was initially used to study muscle function in dogs, this invention laid the groundwork for innovations in cardiorespiratory support. Before their invention, perfusion required interruptions to oxygenate blood from the organ’s vein before transferring it to an arterial reservoir. However, their breakthrough included a double-acting pump utilising an injection syringe, mimicking the heart’s function with two valves that provided pulsatile flow. They also introduced a “preheater” to regulate the arterial blood’s temperature [9].

## Dawn of the 20th century: anticoagulation

**Figure 3:**
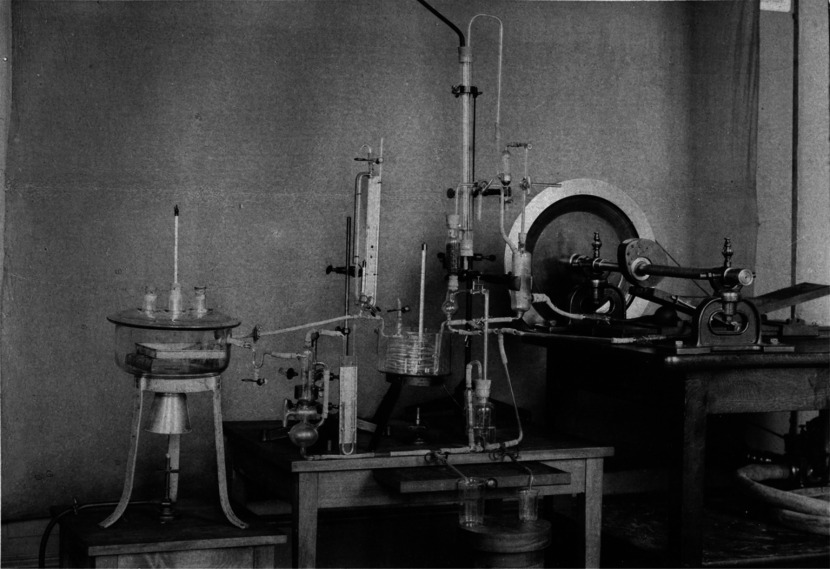
Carl Jacobj’s closed perfusion apparatus (“hematisator”) featured a bubble oxygenator. (Available from the public domain in the USA; downloaded from https://de.m.wikipedia.org/wiki/Datei:Jacobj_Apparat_Foto.jpg.)

Early experiments were limited by the use of defibrinated blood, restricting them to in vitro studies. In 1890, Carl Jacobj developed a closed perfusion apparatus that featured a bubble oxygenator known as the “hematisator” at the Pharmacological Laboratories in Strasbourg (Figure 3). In 1895, Jacobi introduced a ventilated lung to replace the oxygenator, enabling the simultaneous perfusion of both the lung and the organ being examined. Additionally, Jacobj was the first to pharmacologically inhibit coagulation by isolating hirudin from leeches [9]. In 1916, Jay McLean, a second-year medical student at Johns Hopkins University, discovered heparin, enabling continuous anticoagulation. However, heparin was not routinely used until 1931, when the purified form became available [9–12].

## 20th century: initial steps

**Figure 4:**
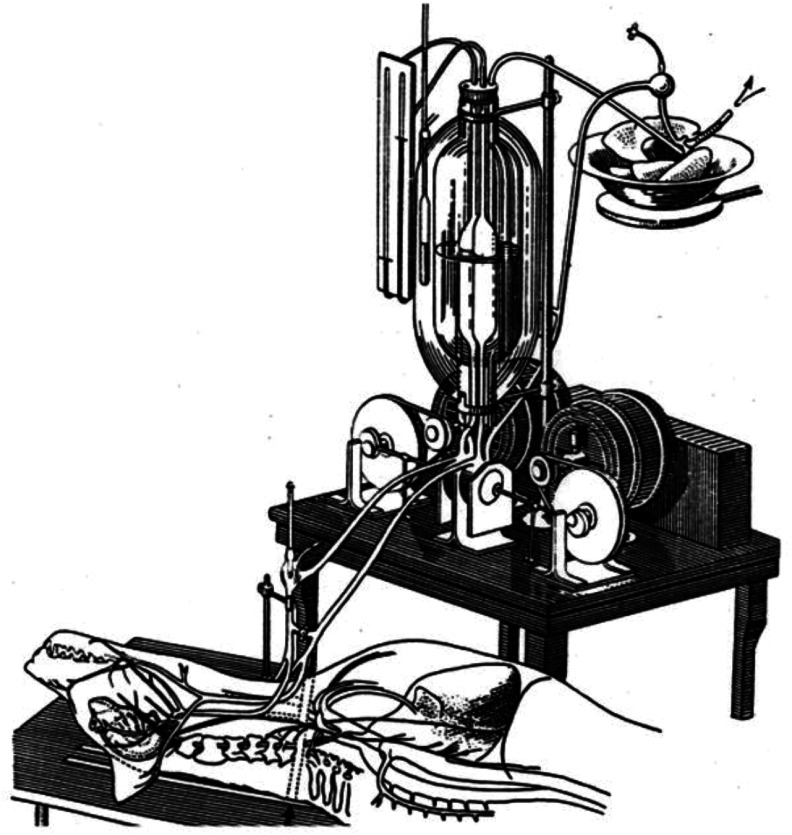
Brukhonenko’s “autojector” was the first documented whole-body extracorporeal circulation using isolated ventilated lungs for gas exchange. (Downloaded from https://commons.wikimedia.org/wiki/File:Patent_autojektor.gif. According to article 1259 of Book IV of the Civil Code of the Russian Federation No. 230-FZ of December 18, 2006, this work is not an object of copyright.)

In 1926, in the Soviet Union, Sergei Sergeevich Brukhonenko stopped a dog’s heart and maintained circulation for two hours using a device called the “autojector” (Figure 4). This marked the first documented whole-body extracorporeal circulation using isolated ventilated lungs for gas exchange [9]. Between 1926 and 1937, Terebinski employed the “autojector” for experimental open-heart valve surgeries on animals. From 1936, a bubble oxygenator developed by Brukhonenko was integrated into these extracorporeal circuits [9].

**Figure 5:**
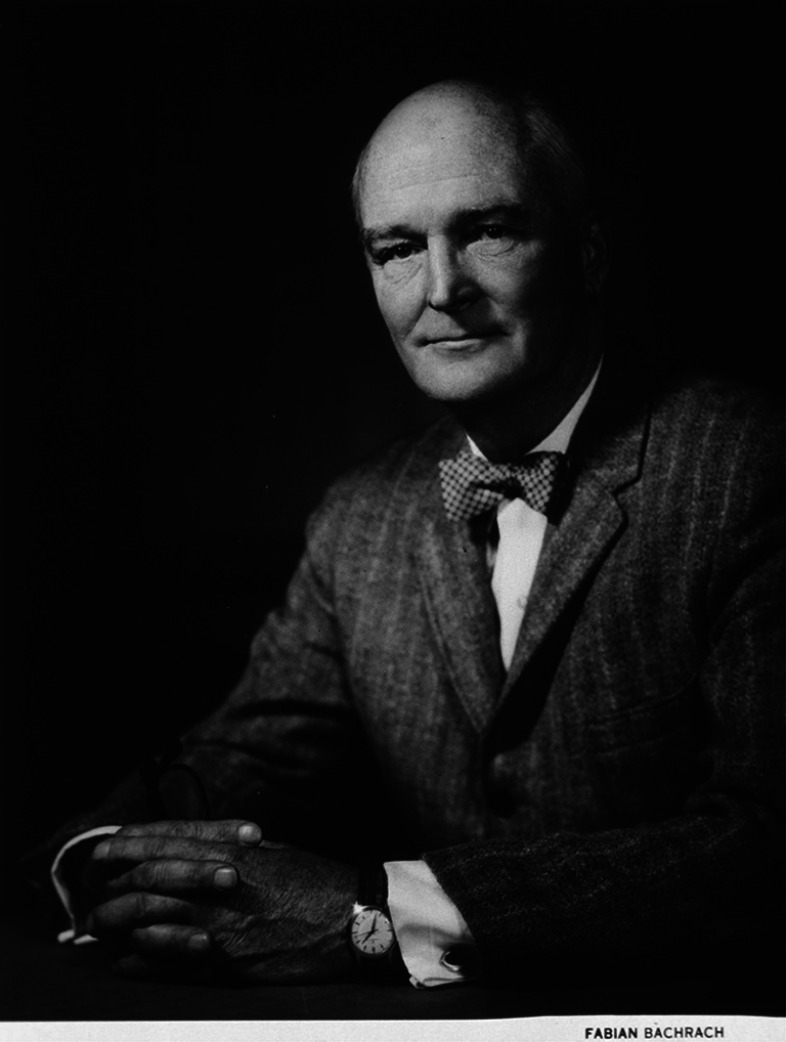
John H. Gibbon Jr. portrait for the 1968 Albert Lasker Clinical Medical Research Award. (Available from the public domain in the USA. Downloaded from https://commons.wikimedia.org/wiki/File:John_H._Gibbon_Jr..jpg.)

Between 1937 and 1953, John Heysham Gibbon (Figure 5) in Philadelphia developed his film oxygenator through animal experiments. It consisted of wire mesh screens arranged vertically and parallel in a plastic container through which blood flowed, forming a stable film that was exposed to a flow of oxygen [13]. This design contributed to creating the Mayo-Gibbon pump-oxygenators [14]. Meanwhile, in the 1940s, Clarence Crafoord and Viking Olov Björk from Stockholm devised their rotating disc oxygenators [9, 11, 14].

## Introduction to clinical practice: from the 1950s to the 1990s

**Figure 6:**
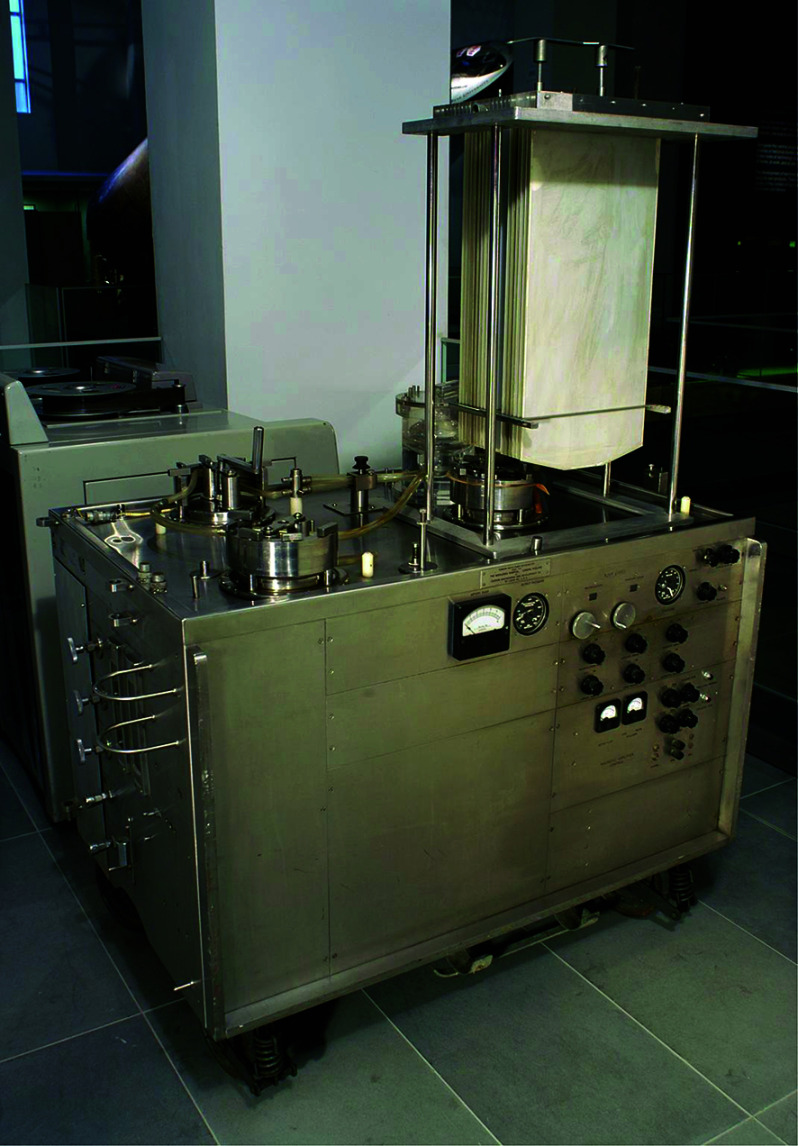
Gibbon-Mayo pump oxygenator (screen type) heart-lung machine. (Science Museum Group. Gibbon-Mayo pump oxygenator (screen type) heart-lung machine. 2000-71 Science Museum Group Collection Online. Accessed 24 November 2024. https://collection.sciencemuseumgroup.org.uk/objects/co523869/gibbon-mayo-pump-oxygenator-screen-type-heart-lung-machine.)

The first clinical use of extracorporeal technology occurred in 1951 when Mario Dogliotti, in Turin, Italy, performed a partial right heart bypass with a bubble oxygenator in a patient to resect a mediastinal tumour successfully [9, 10]. Another milestone was marked on April 5, 1951, when Clarence Dennis attempted to repair a congenital defect in a 6-year-old girl using CPB. Unfortunately, this patient did not survive the procedure. However, Gibbon, in collaboration with International Business Machines (IBM) Corporation, continued developing his cardiopulmonary bypass machine featuring a vertical screen oxygenator (Figure 6), and the first successful cardiac procedure using CPB to correct an atrial septal defect in an 18-year-old patient was performed by Gibbon in Philadelphia on May 6, 1953 [9, 10]. However, despite this inaugural success, Gibbon lost three out of his four congenital patients. Moreover, by 1953, many successful operations had been performed to close atrial septal defects using hypothermia and inflow occlusion or employing the “Gross well” technique [15]. As a result, he decided to abandon open-heart surgery, considering himself more of a scientist and scholar rather than a pioneering surgeon. True to this perspective, he did not actively seek recognition for his contributions [15].

The initial outcomes of cardiac operations using CPB were bleak. Clarence Walton Lillehei from Minneapolis, Minnesota, USA, reviewed open-heart surgeries using CPB performed in six centres between 1951 and 1955. He found that 17 patients died, and only one survived out of 18 operations due to various CPB-related complications [15]. Consequently, alternative experimental approaches were pursued.

In 1954, William T. Mustard, a young paediatric orthopaedic surgeon in Toronto, operated on seven children with congenital heart disease using “Cowan Perfusion Pumps” and Rhesus monkey lungs, which were suspended in a glass flask and utilised as oxygenators [15, 16]. Tragically, all seven patients died either immediately or shortly after their procedures. Despite these devastating outcomes, Mustard felt “*this application of an extracorporeal circulation could be undertaken in less hopeless cases*” [16].

In 1957, Lillehei reported on 45 paediatric patients who underwent congenital cardiac operations between 1954 and 1955 using “Controlled Cross Circulation” [17]. This technique involved cannulating healthy parent donors via their femoral vessels, and the donor circulation provided oxygenated blood for the patients. External pumps were incorporated into the circuit to regulate blood flow. Of the 45 patients, 26 survived the intervention. Notably, the survival rate exceeded that of mechanical CPBs and oxygenators, defying a critic’s prediction of “*200% mortality*” (patient and donor) [15]. Lillehei concluded that “*both in theory and in practice, it is unlikely that a technique for total cardiopulmonary bypass will be developed which, for the patient’s safety, possesses more advantages than this one*” [17].

Nevertheless, Lillehei also reported 305 open-heart operations using CPB between 1954 and 1957, with an improved survival rate of 67%. During these procedures, they utilised a polyvinyl bubble oxygenator and Sigmamotor pump, borrowed from the dairy industry, to drive the CPB machine [15, 17]. In 1959, Charles Drew at Westminster Hospital in London reported three congenital paediatric cases in which he utilised left and right bypass circuits with the patient’s lungs as oxygenators and deep hypothermic circulatory arrest, resulting in the survival of two children [15, 18].

### Technical innovations

**Figure 7:**
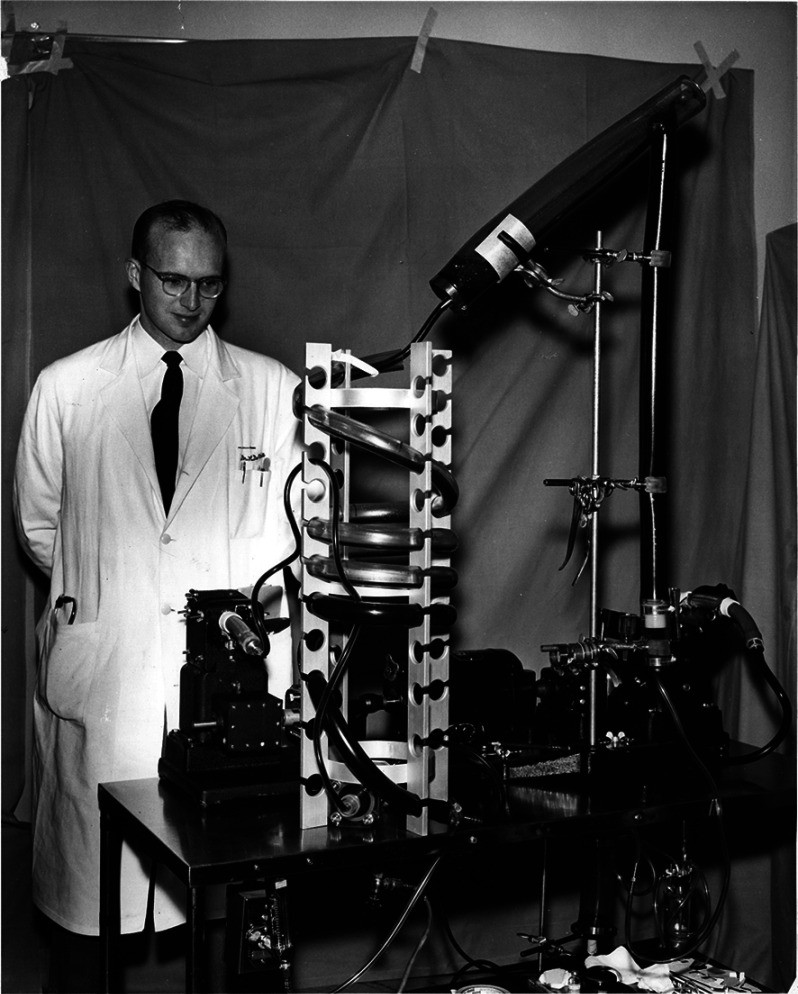
Dr. Richard A. DeWall with his bubble oxygenator, University of Minnesota. (Courtesy of University of Minnesota Archives, University of Minnesota – Twin Cities.)

During the 1950s and 1960s, significant advancements in CPB technology emerged, particularly in the development of oxygenators, cannulation techniques, and other innovations in cardiac surgery [10]. Clarke, Gollan, and Gupta developed their bubble oxygenator in 1952, which evolved into the DeWall “sequential bubble oxygenator” in 1956 (Figure 7) [14, 19]. Bubble oxygenators introduced oxygen directly into the blood, forming bubbles that facilitated gas exchange but posed risks such as air embolism, impaired blood homeostasis, and limited gas exchange duration [13]. In 1958, D.A. Cooley designed a commercially successful disposable bubble oxygenator at the Texas Heart Institute, manufactured by Baxter Travenol Corporation (McGaw Park, IL, USA) [20]. This simple oxygenator was used even until the early years of the 21st century [13].

A significant advancement was the introduction of a large surface area semi-permeable membrane between the oxygen and blood, eliminating direct blood-oxygen contact and enabling longer extracorporeal support. Membrane oxygenators became the most atraumatic type of oxygenator. They do not require a gas removal system and present a low risk of gas embolism [13]. In 1955, when the modern roller pump design was introduced for cardiovascular surgeries, Kolff developed the first membrane oxygenator using wrapped-around polyethylene tubing [21]. In 1956, Clowes and Neville developed a flat, plate-type Teflon membrane oxygenator for cardiac surgery [13, 21]. In 1963, Kolobow constructed a coiled oxygenator with a silicone rubber envelope wrapped around a nylon knit, dramatically improving the gas exchange capability [22]. In 1971, Kolobow developed his disposable coil membrane oxygenator using silicone rubber to improve the oxygenator membrane structure [22]. His oxygenator became the only available oxygenator for ECMO support and was used by Hill et al. in 1971 for the first successful respiratory ECMO support [13].

Since the 1970s, modern hollow-fibre oxygenators have become the standard of care. The first microporous hollow-fibre membrane oxygenator was developed by Y. Nose in 1972. However, the first commercially available hollow-fibre silicone-coated microporous polypropylene (PP) oxygenator was the Capiox, developed by Kozo Suma and the Terumo Corporation in Tokyo, Japan, in 1981 [13]. Although silicone hollow fibres initially faced coagulation issues, implementing silicon-coated microporous PP hollow fibres has significantly reduced these problems while improving gas transport, representing a significant breakthrough [23]. These improvements laid the foundation for the later development of ECMO, which, despite sharing some similarities with CPB, has distinct differences that will be discussed later. Nevertheless, the initial ECMO devices were built upon CPB circuits.

### ECMO gains independence

**Figure 8:**
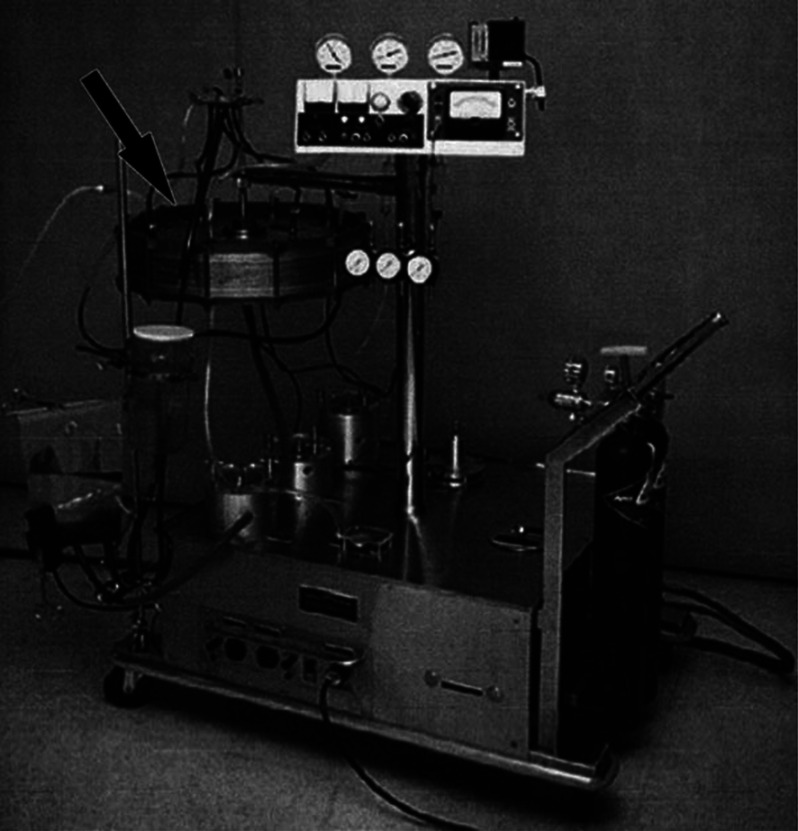
The Bramson heart-lung machine. (Adapted from Yeager and Roy, 2017 [23]. With permission from John Wiley and Sons, the license number is 5936590898239.)

**Figure 9:**
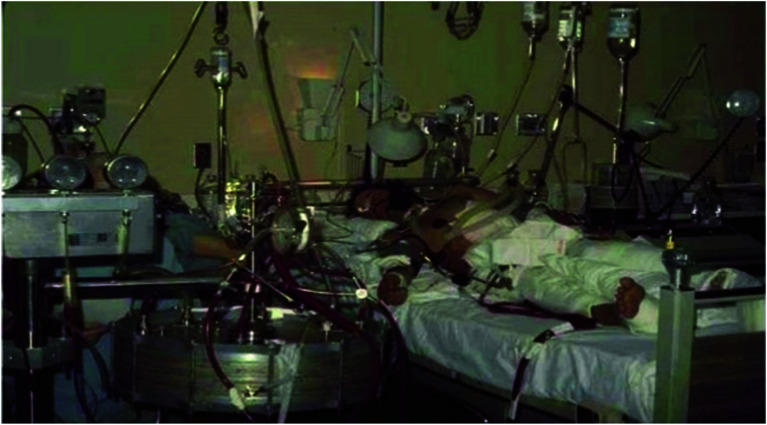
The first successful ECMO patient treated by J. Donald Hill used the Bramson oxygenator. (Adapted from Bonacchi M. Extracorporeal life support in polytraumatized patients. Int J Surg. 2016;33(Pt B):213–217 [27]. With permission from Elsevier, the license number is 5936590106152.)

ECMO branched from other extracorporeal support early. In 1957, George Clowes attempted to use venoarterial (VA) extracorporeal support with an oxygenator to rescue two patients who had suffered cardiac arrests at the Cleveland Clinic in Ohio. Although the attempt was unsuccessful, this event marked the first instance of extracorporeal cardiopulmonary resuscitation (ECPR) [24]. Subsequent developments in oxygenator designs, including dimethylpolysiloxane membranes and the Bramson membrane, improved the suitability of oxygenators for extended use (Figure 8). The progress was aided by a series of animal experimentation and, in some cases, ethically questionable human experiments, such as Rashkind’s work on moribund infants and animals [24, 25]. In 1969, Hill et al. from San Francisco, California, presented a case series involving VA and VV (venovenous) extracorporeal supports for cardiac and respiratory failure patients. Unfortunately, survival was not achieved in these early cases [26]. However, in 1971, the same team in Santa Barbara (California, USA) achieved a breakthrough by successfully treating a 24-year-old trauma patient with respiratory failure using peripheral VA extracorporeal support and a Bramson membrane heart-lung machine (Figure 9) [1, 27]. This marked the first successful clinical application of ECMO; however, the word “ECMO” was not used in the original publication [1]. The inaugural success in neonatal ECMO treatment was marked by “Baby Esperanza” (meaning “hope” in Spanish), a newborn girl battling meconium aspiration and severe cardiorespiratory failure in 1975. In Ann Arbor, Michigan, Bartlett et al. utilised venoarterial ECMO (VA-ECMO) to manage her cardiorespiratory failure and subsequently ligated the girl’s patent ductus arteriosus. Remarkably, after 6 days, the ECMO support was successfully withdrawn, and the patient fully recovered, eventually becoming a mother of two [22, 28, 29].

Modern hollow-fibre oxygenators from the 1970s enabled longer ECMO runs, but “oxygenator wetting” was still a concern. This resulted in blood plasma slowly infiltrating micropores, greatly hindering gas transfer and shortening the oxygenator’s lifespan. Often, two to three oxygenators were needed for one ECMO patient [12]. However, the problem was addressed using “crossflow” hollow-fibre, non-microporous oxygenators and a new membrane material, polymethylpentene (PMP), introduced in the early 21st century, marking a giant leap forward [23, 30]. The evolution of oxygenator designs over three decades was truly remarkable, progressing from the first-generation flat membrane PDMS (polydimethylsiloxane) oxygenators of the 1970s to the second-generation microporous PP hollow-fibre oxygenators of the 1980s, and culminating in the advanced third-generation oxygenators featuring modern non-microporous PMP membranes. Oxygenators became more durable, providing high gas permeability with minimal blood leakage [30, 31].

**Figure 10:**
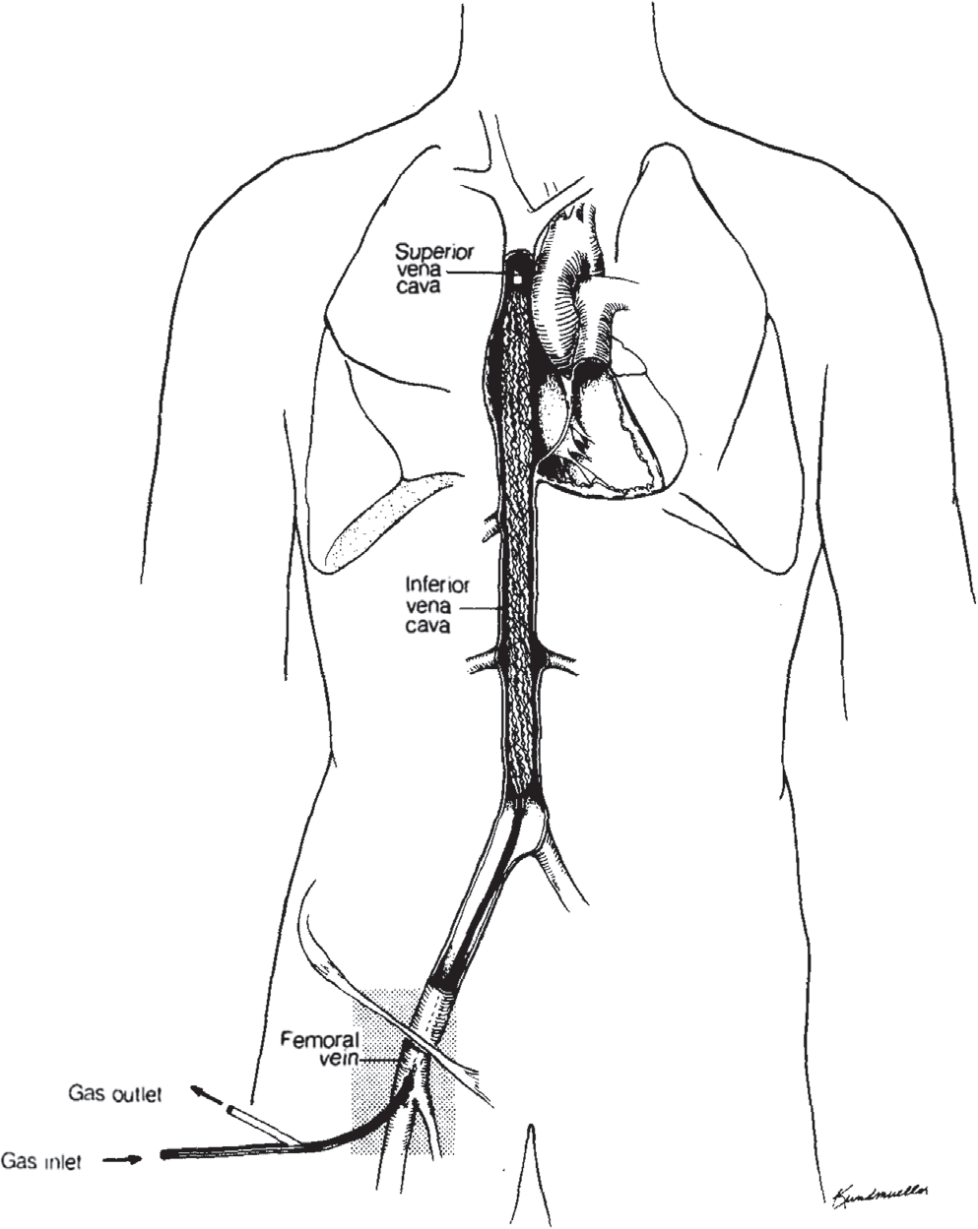
IVOX: Intravascular Oxygenator. (Adapted from Mortensen, 1992 [33]. With permission from John Wiley and Sons, the license number is 6022060775627.)

A noteworthy research endeavour from the 1980s was the implantable intravascular oxygenation system. In 1987, J.D. Mortensen from Salt Lake City, Utah, USA, published a preliminary report on an Intravenacaval Blood Gas Exchange (IVCBGE) device [32], rebranded later as IVOX (Intravascular Oxygenator) [33]. IVOX featured a hollow-fibre flexible oxygenator with a slim gas inlet and outlet pipe (Figure 10), allowing bedside, ambulance, or field insertion into a central vein, achieved percutaneously or via venous cut-down. While this technology represented a promising approach and spurred worldwide research [32–42], it ultimately did not succeed. Although IVOX reduced ventilatory support by at least 25% in about 50% of patients [34], it proved considerably less efficient than modern ECMO devices. In addition to limited gas transfer, 29.3% of IVOX devices had reported mechanical and/or performance issues [34]. Blood or gas leakages and bubble formations were also problematic [35]. Despite these challenges and the absence of broad clinical adoption, research has persisted into recent years. In their 2022 review, Straube et al. documented a dozen experimental intravascular gas exchange devices, including their own experimental innovation [35].

### Evidence-based practice and organisational development in the late 20th century

The first prospective, randomised ECMO trial to manage severe acute respiratory failure in adults was conducted in the USA in 1975. The results were published in 1979, establishing the term “*extracorporeal membrane oxygenation (ECMO)*” [43]. Unfortunately, the trial had significant design and execution flaws, leading to discouraging outcomes. Survival rates were below 10% in both the trial’s arms (ECMO vs. conventional ventilation), which hindered the widespread adoption of ECMO technology in adult practice for almost two decades [43, 44]. Interest in ECMO was revived in 1978 with the introduction of the ECCO_2_R technique by Kolobow et al. and Gattinoni et al., which was found to be less harmful [14, 45–48].

A subsequent discouraging trial was conducted by Morris and colleagues in 1994, investigating extracorporeal carbon dioxide removal versus inverse ratio ventilation in acute respiratory distress syndrome (ARDS) [49]. The authors did not recommend extracorporeal support for the management of ARDS [49]. Although the term ECMO was used in their publication, their device significantly differed from modern ECMO devices of the 21st century.

Nevertheless, the results were more encouraging in paediatric practice. Randomised controlled trials by Bartlett et al. in 1985 and O’Rourke et al. in 1989 demonstrated survival benefits with ECMO compared to conventional therapies [50, 51]. By 1986, 18 neonatal centres in the USA had established ECMO teams [52]. In a paediatric study conducted in 1982 [53] and a registry report published in 1988 [52], Bartlett et al. [53] and Toomasian et al. [52] suggested that ECMO could be effective in adults if applied before irreversible lung damage occurs [52, 53]. Over the following decades, this key suggestion became an essential criterion for accepting patients for ECMO, intended to “bridge to recovery.”

In 1993, Schumacher et al. published a randomised trial with a cost-benefit analysis, concluding that paediatric ECMO did not increase hospital stay and costs; additionally, early ECMO might offer morbidity benefits [54]. In 1996, a UK collaborative randomised trial demonstrated the clinical effectiveness of a well-staffed and organised neonatal ECMO service [55]. In the 1990s, neonatal ECMO achieved an overall survival rate of 85%. The number of ECMO cases peaked during this period, with the subsequent gradual decrease attributed to improved conventional ventilation strategies and better treatment options [44].

A significant milestone was the establishment of the Extracorporeal Life Support Organization (ELSO) in 1989 in New Orleans [14]. ELSO collects global data, publishes guidelines and books, and offers education through courses, conferences, webinars, and online training. This international organisation unites adult and paediatric clinicians, academics, professionals, and industry representatives to improve extracorporeal technology. As of September 2024, the ELSO directory includes member centres from 68 countries [56].

## 21st century: worldwide establishment of ECMO in clinical practice and research

In the new century, the success achieved in neonatal practice and advancements in device designs motivated clinicians in adult practice, leading to the ground-breaking CESAR (Conventional ventilatory support vs. Extracorporeal membrane oxygenation for Severe Adult Respiratory failure) trial in 2009 [57]. This trial demonstrated the survival benefit of ECMO over conventional ventilation in carefully selected adult ARDS patients, emphasising the importance of initiating ECMO before irreversible lung damage occurs [57]. In the same year, amidst the H1N1 influenza pandemic, global health services experienced considerable strain. As a result, ECMO emerged as a valuable tool for managing patients with respiratory failure unresponsive to conventional ventilation. This was demonstrated by the almost 80% ECMO survival and 71% overall H1N1 survival rate in a study by Davies and colleagues from Australia [58]. Following the encouraging results of the CESAR trial, six national ECMO centres were established in the UK in 2011, growing to eight centres subsequently. Over time, these centres have gained substantial expertise, managing increasingly extended and complex ECMO cases, ultimately enabling the survival of critically ill patients, often in extreme conditions. Consolidating resources within a nationally coordinated network of specialised centres has played a pivotal role in achieving this success [59].

In 2018, Combes et al. published the EOLIA (ECMO to Rescue Lung Injury in Severe ARDS) trial [60], revealing no statistically significant survival advantage at 60 days between ECMO and conventional ventilation in ARDS. The interpretation of these results has triggered some debates. Notably, the study was acknowledged to be underpowered and prematurely terminated. It also involved patients from smaller centres with less experience and utilised ECMO as a rescue measure for the control arm [60]. In 2020, the outbreak of the COVID-19 (coronavirus disease 2019) pandemic resulted in a substantial surge in ARDS cases, generating an overwhelming need for ECMO. To address this demand, the UK national capacity for ECMO was augmented, and emergency protocols were implemented. As a result, clinicians accumulated enhanced expertise during this period, refining ECMO care practices. This translated into improved patient selection, often extended ECMO durations and improved survival rates. These advancements also prompted updates in society recommendations [61].

### Current trends

#### Improved oxygenator and circuit design

Modern hollow-fibre oxygenators with enhanced gas exchange capabilities have become the standard for ECMO care. Circuit prime volumes have decreased (<300 mL), and heat exchangers have been incorporated into the oxygenator design. Newer, more durable oxygenator designs allow smoother blood flow, minimising shear stress. Biocompatible surface coatings and integrated arterial line filtration have improved complication rates, making ECMO runs safer and longer. A significant advancement is the reduced surface area of oxygenators, which minimises blood contact with artificial surfaces and decreases the risks of thrombosis and haemolysis. These innovations minimise cell damage, consumption, and platelet adhesion [62, 63].

Centrifugal pumps have generally replaced roller pumps in modern ECMO care due to their simpler operating requirements and reduced damage to blood components [64]. The latest designs incorporate magnetic levitation technology, minimising friction and generating heat [65, 66]. However, recent retrospective database studies challenge the morbidity and mortality benefits of centrifugal pumps compared to traditional roller pumps in the paediatric ECMO population [67, 68].

**Figure 11:**
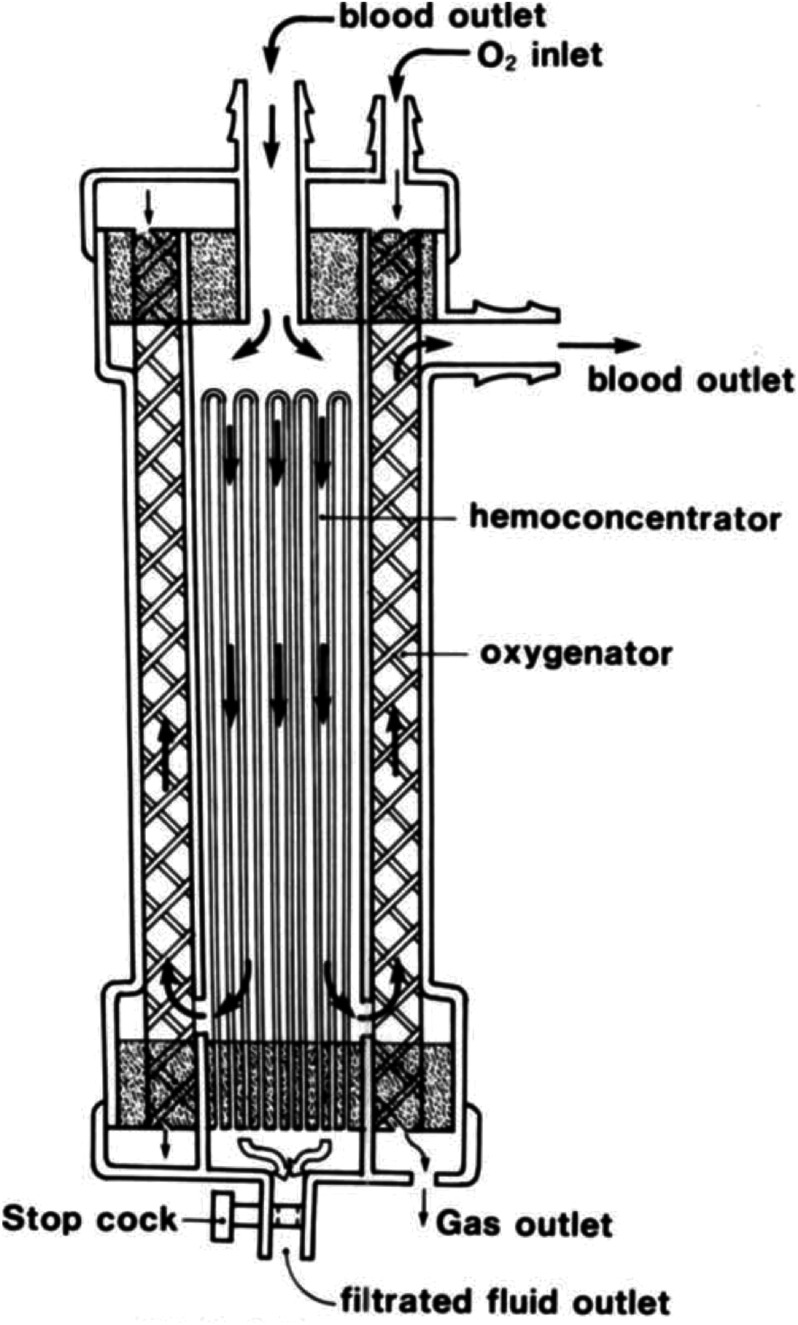
Oxygenator with built-in hemoconcentrator. Nishida et al.’s prototype number 3. (Adapted from Nishida et al., 1995 [75]. With permission from John Wiley and Sons, the license number is 6022670778767.)

Current designs of the ECMO circuit enable continuous renal replacement therapies (CRRT) to be delivered directly through the circuit. One less expensive, albeit imprecise, approach to facilitate CRRT is integrating an inline haemofilter within the ECMO circuit via a shunt, without using a separate CRRT device [69–71]. However, adjusting the required flow rates could be cumbersome, risking haemolysis and thrombus formation [69, 70]. The other, more popular method combines the two circuits when a CRRT device is integrated into the ECMO circuit [69–71]. This approach offers enhanced stable control over the blood flows of ECMO and CRRT devices as well as precise fluid management [69–71]. Haemoadsorption filters could similarly be integrated into ECMO circuits; however, the existing evidence regarding their benefits remains contentious, necessitating further research [72]. CRRT machines equipped with integrated PrismaLung+ devices are also suitable for low-flow extracorporeal carbon dioxide removal [73, 74]. Despite their resemblance to ECMO – comprising a blood pump and a CO_2_ removal filter – these devices cannot oxygenate patients [73, 74], and their carbon dioxide removal efficiency is lower than that of high-capacity ECMO systems. To integrate CRRT and oxygenator functions, Nishida et al. conducted experiments in 1995 and 1997, in which they incorporated haemofilters (“hemoconcentrators”) into oxygenators (Figure 11) [75, 76]. While their laboratory evaluations yielded promising results, this concept has not been widely adopted in clinical practice.

Flexible and durable circuit and cannula designs enable ambulatory ECMO, which is particularly transformative, allowing patients with severe cardiopulmonary conditions more freedom to move around and participate in daily activities, significantly improving their strength and quality of life and reducing healthcare costs [77–79]. Before lung or heart transplant, awake ECMO or other ECLS forms are increasingly chosen over conventional mechanical ventilation. However, deploying ambulatory ECMO requires careful monitoring of anticoagulation levels, circuit safety, and comprehensive education for patients and caregivers. This strategy helps prevent complications associated with prolonged intubation, such as ventilator-induced lung injury and infections [78, 79]. While initial studies are promising, further research is necessary to fully confirm the benefits of this innovative approach. The discussion over whether two distinct, specially designed cannulae – e.g. with lighthouse tip return lines [80–82] – or dual-lumen cannulae – e.g., Avalon [83] – offer greater advantages continues to be debated at conferences and in research studies.

A particularly innovative dual-lumen device is the ProtekDuo cannula (LivaNova, London, UK) [84, 85], which is a percutaneous right ventricular mechanical assist device designed to support right ventricular failure of diverse aetiologies [85]. Additionally, incorporating an oxygenator into the circuit can provide concurrent ECMO respiratory support without oxygenated blood recirculation [85, 86]. While the survival benefit compared to other temporary percutaneous configurations or devices has yet to be established [87], ProtekDuo’s innovative dual-lumen design makes it a versatile tool [85–87]. The cannula is introduced percutaneously and coupled to an external centrifugal pump, e.g. the TandemHeart (LivaNova, London, UK), LifeSPARC (LivaNova, London, UK) or CentriMag (Abbott, Pleasanton, CA, USA) [85, 86]. It resembles a thicker pulmonary arterial catheter that drains blood from the right atrium and returns it to the pulmonary artery, effectively bypassing the right ventricle. Its groin-sparing access permits early mobilisation, while compatibility with a wide range of pumps and oxygenators allows the device to be incorporated into multiple circuit configurations [85, 86].

The ProtekDuo can be paired with durable left ventricular assist devices to deliver early, postoperative biventricular circulatory support [85, 86]. For temporary biventricular assistance, it may also be combined with the Impella device (Abiomed Inc., Danvers, Massachusetts, USA) in the configuration known as “ECPella 2.0” or “PROPella” [85, 86, 88, 89]. While the ProtekDuo has been successfully explored for other indications, such as drainage during VA-ECMO in lung transplantation and isolated transapical left ventricular support, these applications are currently supported by only a limited number of case reports [85, 86].

#### Mechanical circulatory support and extracorporeal cardiopulmonary resuscitation

Venoarterial ECMO (VA-ECMO) has become an essential modality for short-term mechanical circulatory support, providing critically ill patients with the necessary time to recover, undergo definitive surgical intervention, receive a durable ventricular assist device (VAD), or await donor heart availability for transplantation [90–96]. Typical VA-ECMO indications for bridge to non-transplant cardiac surgery may include postinfarction ventricular septal or papillary muscle ruptures, traumatic injury of the heart and chronic thromboembolic pulmonary hypertension [92]. In the post-cardiotomy setting, VA-ECMO can be instituted directly in the theatre when low cardiac output syndrome or failure to wean from cardiopulmonary bypass threatens imminent cardiovascular collapse, gaining crucial hours or days for myocardial reperfusion, ventricular rest and recovery. In such cases, VA-ECMO typically remains central, with right atrial drainage and aortic return flow [93]. However, peripheral surgical cannulations are also utilised, including femoro-femoral and femoro-axillary approaches (where venous drainage occurs via the femoral veins and arterial return through the axillary arteries) [90, 92, 93, 96]. Femoral arterial cannulations typically require limb reperfusion cannula insertion to prevent limb ischaemia.

Central ECMO unloads both ventricles, reduces the need for high-dose catecholamines and allows a period, often several days, for the stunned myocardium to regain strength [90, 91, 95]. Additionally, central return cannulations may reduce the likelihood of competitive retrograde flow and associated hypoxia [93, 95, 97, 98]. However, peripheral configurations may require additional retrograde trans-aortic left ventricular unloading due to retrograde flow. This can be achieved using iVAC or Impella (Abiomed Inc., Danvers, Massachusetts, USA) devices [91]. The concurrent use of Impella (Abiomed Inc., Danvers, Massachusetts, USA) and VA-ECMO is referred to as “ECPella” or, more recently, “ECPella 5+”, featuring a higher-capacity Impella device that can efficiently unload the left ventricle [99–101].

ECMO technology enabled ECPR, extending the temporal salvage window following out-of-hospital and in-hospital cardiac arrests. Conventional cardiopulmonary resuscitation can provide only 25–30% of cardiac output, whereas ECPR can restore the entire cardiac output, ensuring sufficient organ perfusion [97, 98, 102]. According to Wengenmayer et al.’s recent review, this approach has contributed to 20–43% hospital survival rates and meaningful neurological recovery in 14–30% of patients who might otherwise have been lost [97]. For out-of-hospital cardiac arrests, ECPR typically involves femoro-femoral cannulation either at the scene, in ECMO centre emergency departments [97], or, in cases of greater geographical distance, at an intermediate initiation hospital (“Minnesota model”) [97, 103]. Establishing mobile ECMO teams, optimal patient selection, structured pathways and protocols, and developing specialised ECPR kits are essential [97, 98, 102, 103].

Once haemodynamic stability is secured, the same circuit can be rearranged in situ: removal of the oxygenator converts the system to an isolated right-ventricular assist device (RVAD); redirection of the arterial limb to the ascending aorta, coupled with left-atrial drainage, fashions an interim left-ventricular assist device (LVAD); or dual drainage with dual return yields a fully percutaneous biventricular (BiVAD) arrangement. Such modularity allows seamless progression from emergent ECMO to intermediate VAD support while avoiding haemodynamic “blackouts” associated with circuit exchanges [104]. Staged weaning trials under echocardiographic surveillance can culminate in complete decannulation for patients whose ventricles recover. For those with irreversible cardiomyopathy, however, ECMO buys crucial time to assess candidacy for durable centrifugal pump VAD implantation or to secure a donor heart [95].

The TandemHeart (LivaNova, London, UK) is a percutaneous ventricular assist device utilising an external continuous-flow centrifugal pump without oxygenators [91, 105, 106]. For left-sided support, oxygenated blood is withdrawn from the left atrium via trans-septal puncture and returned to the systemic circulation through the femoral artery, bypassing the native left heart. Right-sided support is achieved by positioning the drainage cannula in the right atrium and the return cannula in the pulmonary artery. However, widespread adoption remained limited due to the necessity of trans-septal access, alongside an elevated risk of limb ischaemia and coagulopathy. Furthermore, its use has not reduced overall mortality [91, 105].

Given the already substantial length and specific focus of this review, a more detailed discussion of mechanical circulatory support lies beyond its scope.

#### Miniaturisation of ECMO equipment

Historically, ECMO machines were bulky and required substantial space in hospitals. However, recent advances in miniaturisation have significantly transformed ECMO therapy by enhancing its portability and flexibility. Modern ECMO systems are now more compact, equipped with handles and wheels, allowing for rapid deployment at the patient’s bedside, which is crucial during emergencies like cardiac arrest. This flexibility enables ECMO support within hospitals and across different facilities, providing swift and essential cardiorespiratory support to patients during mass casualty incidents. This adaptability has expanded ECMO’s use beyond intensive care units.

For instance, the Cardiohelp (Maquet Getinge Group, Rastatt, Germany) and CentriMag (Abbott, Pleasanton, CA, USA) systems offer versatile ECMO and VAD support that is easily transportable across medical facilities [107, 108]. The Novalung system (Fresenius Medical Care, Bad Homburg, Germany), tailored for neonates and children, provides precise control over blood flow and oxygenation, which is essential for paediatric care [109, 110]. The Mobybox System (Hemovent GmbH, Aachen, Germany) offers a pneumatically powered ECMO device with an innovative bi-ventricular displacement pump, which does not require an electrical power source and is ideal for situations when limited infrastructure is available, for example, in emergencies in the field or during transport [111].

## Future directions in ECMO

ECMO technology continues to evolve, and several exciting advancements are shaping the future of critical care, cardiovascular surgery, and extracorporeal life support.

### Surface coatings and endothelialisation of ECMO circuits and oxygenators

**Figure 12:**
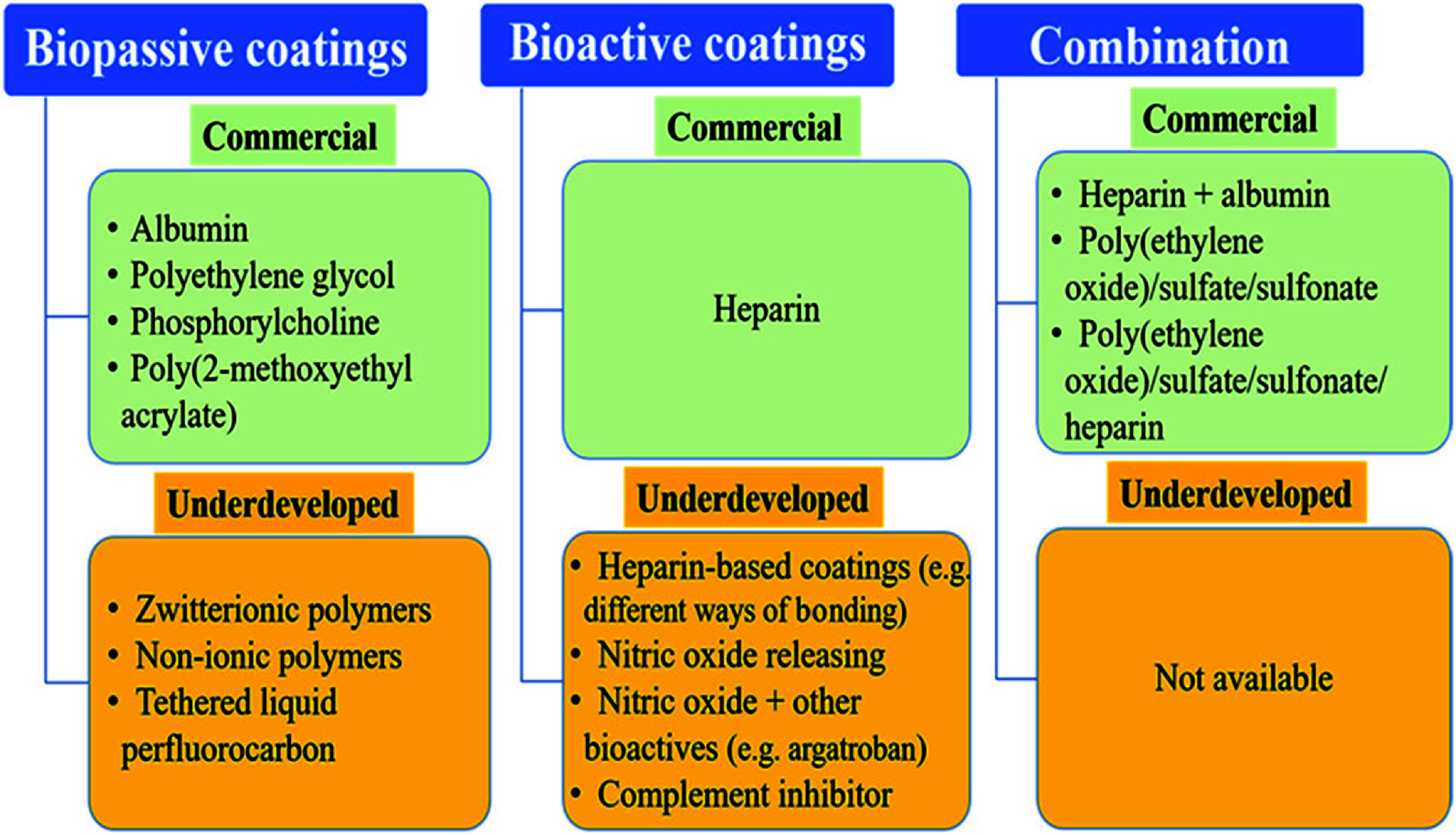
Overview of currently commercial and underdeveloped anti-thrombogenic surface coatings for ECMO. (Adopted from Zhang et al., 2021 [113]. Copyright *©* 2021, American Chemical Society. With permission of ACS Biomaterials Science & Engineering.)

Improved haemocompatibility can be achieved with various circuit and oxygenator inner surface modifications, such as “biomimetic” or “bioactive” surfaces (currently heparin-coated or potential direct thrombin inhibitor coated surfaces, and nitric oxide-releasing surfaces in the future), “biopassive” (non-thrombogenic) surfaces (e.g., phosphorylcholine, albumin and poly-2-methoxyethyl acrylate or other experimental omniphobic (fluid-repellent) surfaces and tethered liquid perfluorocarbon coatings) [31, 112, 113]. Figure 12 summarises these technologies [113]. A detailed evaluation of these technologies is beyond the scope of this historical review due to their complexity, experimental nature, and technical challenges. However, a brief overview of key trends may still be of interest.

In 1963, Gott et al. reported their experiment on heparin-coated surfaces [114]. This “ionic heparin binding” technique, however, exhibited a tendency to swell due to heparin leaching, a phenomenon that was later absent in the “covalently bonded heparin” technology [115, 116]. Heparin bonding was primarily investigated in CPB circuits, where it reduced cellular activation and the release of inflammatory proteins, potentially offering clinical benefits [112]. However, heparin-induced thrombocytopenia has remained a concern despite conflicting results in the literature [117, 118]. Direct thrombin inhibitor coating (e.g. argatroban or bivalirudin) may mitigate this issue [119–121].

**Figure 13:**
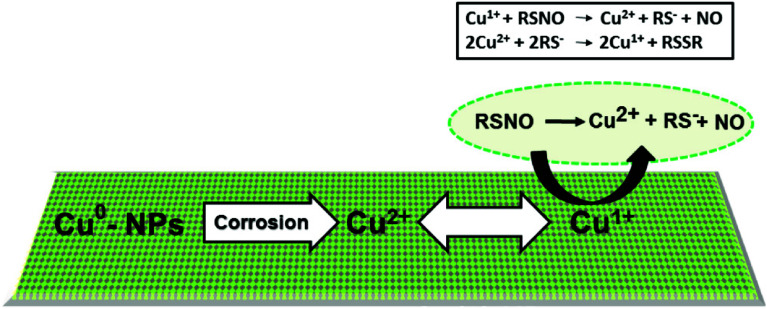
Schematic representation of the mechanism of NO release from an RSNO such as SNAP. The mechanism involves the oxidation of Cu^0^ nanoparticles to Cu^2+^ ions in the presence of water. NO, nitric oxide; SNAP, S-nitroso-N-acetylpenicillamine; Cu, copper; NP, nanoparticle; RSNO, nitrosothiol NO donor; RS^−^, thiolates. (Adopted from Pant J, Goudie MJ, Hopkins SP, Brisbois EJ, Handa H. Tunable Nitric Oxide Release from S-Nitroso-N-acetylpenicillamine via Catalytic Copper Nanoparticles for Biomedical Applications. ACS Appl Mater Interfaces. 2017;9(18):15254–15264 [125]. Copyright *©* 2017, American Chemical Society. With permission from Applied Materials.)

Nitric oxide (NO), an endogenous vasodilator with bactericidal properties, is released by endothelial cells and has both direct and indirect effects in suppressing platelet activation and aggregation [112]. However, NO-releasing surfaces exert only localised effects without causing systemic platelet dysfunction or significant methaemoglobin generation [112, 122]. Various methods are employed to harness NO’s effects in extracorporeal circuits: the first involves mixing NO into the gas of the membrane oxygenator, while the second incorporates NO donors into the polymers of the circuit [112, 119, 123], with the possibility of combining both approaches [124]. However, this technology is constrained by the depletion of NO donor molecules from the circuit surface and the specific manufacturing requirements of the circuits and oxygenators [112]. As a result, new technologies are being explored, such as the use of copper or other metallic-based nanoparticles to improve NO donor integration and NO delivery [112, 125]. Recently, promising tunable NO release has been demonstrated, achieving a 92% reduction in platelet adhesion and inhibition of bacterial adhesion via S-nitroso-N-acetylpenicillamine (SNAP) catalysed by copper nanoparticles in vitro (Figure 13) [125]. Although this technology shows potential, further research and development are required to identify the optimal NO donor molecules for commercial NO-releasing surfaces in extracorporeal circuits and oxygenators.

For “biopassive” surface coatings, relatively thromboresistant phosphorylcholine (PCC), poly-2-methoxyethylacrylate (PMEA), or albumin coatings can be utilised [112, 113]. However, evidence regarding the biocompatibility benefits of coated circuits remains insufficient [126]. Recently, cross-linkable zwitterionic polymer coatings, designed to mimic the cell surface membrane, have demonstrated remarkable improvements in hemocompatibility in vitro. These coatings exhibited significant resistance to fibrinogen adsorption, platelet adhesion, blood cell activation, and thrombus formation; however, further research is needed to clarify these coatings’ stability and potential benefits [113, 127, 128].

**Figure 14:**
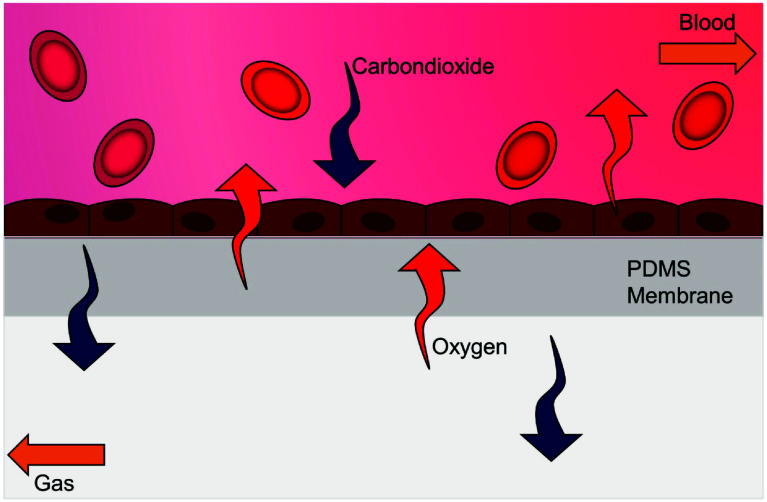
Model of endothelialised membrane oxygenation (EndOxy). Oxygen and carbon dioxide are exchanged between blood and gas via an endothelialised membrane. PDMS, polydimethylsiloxane. (Adopted from Hellmann et al., 2020 [133]. This is an open access article under the terms of the Creative Commons Attribution-Non-Commercial License, which permits use, distribution and reproduction in any medium, provided the original work is properly cited and is not used for commercial purposes. https://creativecommons.org/licenses/by-nc/4.0/. With permission from John Wiley and Sons, the license number is 6032471286131.)

In the field of regenerative medicine, ECMO has been utilised as a platform for delivering stem cells [129–131]. Additionally, the development of biohybrid lungs involves establishing a monolayer of endothelial cells on hollow-fibre oxygenator membrane scaffolds [132]. Interaction between circulating blood and the artificial surfaces within extracorporeal circuits can cause the adsorption of blood proteins and coagulation factors, leading to blood clots, inflammation, and activation of the complement system. These reactions may cause device occlusion and failure of the ECMO system. Endothelialisation technology aims to reduce the contact of circulating blood with the artificial surfaces of the extracorporeal circuits (Figure 14) [133]. This technology is expected to significantly decrease inflammatory reactions and cell damage, ultimately improving outcomes [112, 132].

Endothelisation can be achieved either by pre-seeding the circuit or oxygenator with endothelial cells in vitro (“pre-endothelialisation”) or by inducing in vivo self-endothelialisation with endothelial progenitor cells [112, 126, 132]. Experimental studies have yielded promising early results, including the development of the EndOxy biomimetic oxygenator test device in Germany [133, 134], endothelialisation of titanium dioxide-coated gas-exchange membranes [135] and studies on various alternative surface materials [132]. Nevertheless, efforts to establish a stable, homogeneous single endothelial layer on the gas-exchange surfaces of clinical ECMO equipment remain at an early experimental stage due to the intricate biology of endothelial cells and the technical challenges of coating complex gas-exchange geometries [112, 121, 126].

### Microfluidic oxygenators

Recent research has developed prototypes of microfluidic oxygenators constructed from gas-permeable polydimethylsiloxane. These devices channel blood and oxygen through adjacent micro-channels, which can be arranged in straight lines or branching tree-like patterns. Notably, some designs can use ambient air instead of pure oxygen as the gas source. Although promising, these devices remain experimental and are not yet suitable for clinical use [136].

### Implantable devices

Total artificial lungs (TALs) are envisioned as implantable, pumpless, single-unit ECMO systems. However, recent devices have also been experimental and cannot entirely replace native lung function [136]. Extracorporeal – or rather, paracorporeal – prototypes aimed to preserve patient mobility by relying on right-ventricular propulsion, being attached either in series to the pulmonary artery or in parallel from the pulmonary artery to the left atrium, the latter easing cardiac load but bypassing important metabolic and filtering roles of the natural lungs [137, 138].

In 2002, Chambers et al. reviewed the development of the BioLung (MC3, Inc., Ann Arbor, Michigan) device, which provided 50–60% respiratory support over 30 days in ovine models [136, 138, 139]. However, routing all cardiac output through the device reduced systemic flow by 20–30% [136–138]. To mitigate resistance issues, the compliant thoracic artificial lung (cTAL) device incorporated expandable chambers surrounding a polypropylene fibre bundle. However, animal studies indicated that haemodynamics became unfavourable when more than 60% of cardiac output was diverted [136, 138, 140].

The long-term application of these devices is further constrained by thrombogenicity, necessitating research into enhanced surface coatings, nitric-oxide-releasing PDMS fibres embedded with copper nanoparticles, and pharmacological inhibition of coagulation factor [136, 138]. Total artificial lungs might be the future of artificial lung technology. However, the development required to achieve a fully implantable, pumpless lung replacement remains substantial.

### Artificial intelligence

Another promising direction is the integration of Artificial Intelligence (AI) into ECMO management, which could optimise treatment protocols and predict potential complications (e.g. ECMO PAL) [141], thereby improving overall patient outcomes [141, 142]. AI technology and telemedicine may enable patients to be discharged from the hospital while still on extracorporeal support [22]. Developing more compact ECMO circuits with efficient, biocompatible, and atraumatic oxygenators that require minimal or no anticoagulants could make these innovations possible.

### Ambulatory ECMO

Next-generation ECMO systems may feature closed-loop control where arterial and mixed venous oxygen saturation guide flow adjustments to meet target levels or indicate corrective measures. Servo-regulated pumps can sustain predetermined blood flow and automatically adapt to patient changes, movement, or hypovolemia [143]. Automation and servo‐regulated control could empower bedside nurses to manage routine ECMO care, freeing ECMO specialists to focus on supervisory, educational, and emergency responsibilities. These advancements, along with improved oxygenator designs, progress in new anticoagulants, and remote internet-based monitoring, could facilitate the creation of implantable, miniature or wearable paracorporeal membrane lungs for bridging to transplants or serving as destination therapy in advanced lung disease [143]. After some education, stable patients could be ambulatory, discharged from hospitals and monitored at home under safeguards akin to ventricular-assist‐device programmes [143].

**Figure 15:**
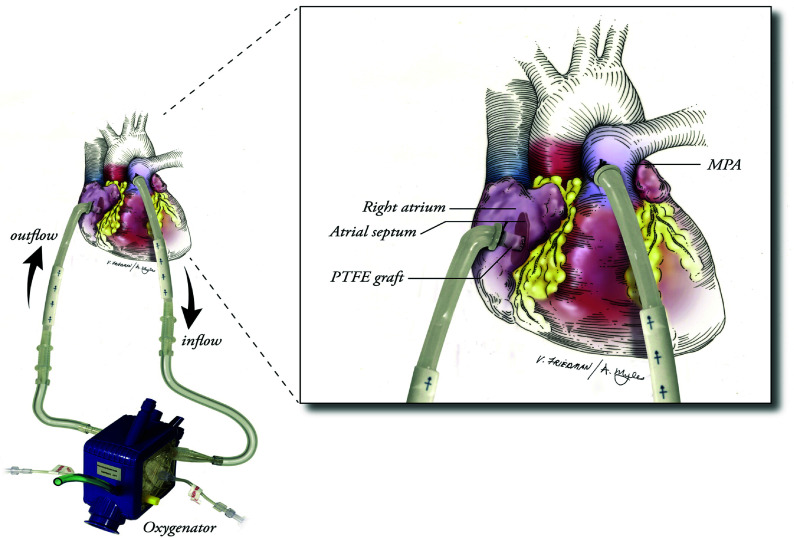
Diagram demonstrating the cannulation of the left atrium for the fourth patient treated with paracorporeal oxygenator support. A 10-mm Gore-Tex graft extension was added to a 6-mm Berlin cannula. The Gore-Tex graft was passed through the right atrium and sewn to the interatrial septum. MPA, Main pulmonary artery; PTFE, polytetrafluoroethylene. (Adopted from Hoganson et al., 2014 [144]. Copyright © 2014, with permission from Elsevier, the license number is 6022010537439.)

These devices might function passively, driven by pulmonary or systemic arterial pressure, or be configured from the pulmonary artery to the left atrium, unloading the right ventricle while providing full oxygenation and CO_2_ clearance [143]. One example of a paracorporeal lung assist device was published in 2014 by Hoganson et al. [144] (Figure 15). They transitioned four paediatric patients awaiting lung transplantation from ECMO to a pumpless paracorporeal lung assist device [144]. Although two patients died due to complications, such as haemorrhagic stroke, cardiac failure, and renal failure, which prevented lung transplantation, their cases nonetheless demonstrated the feasibility of the concept [144].

**Figure 16:**
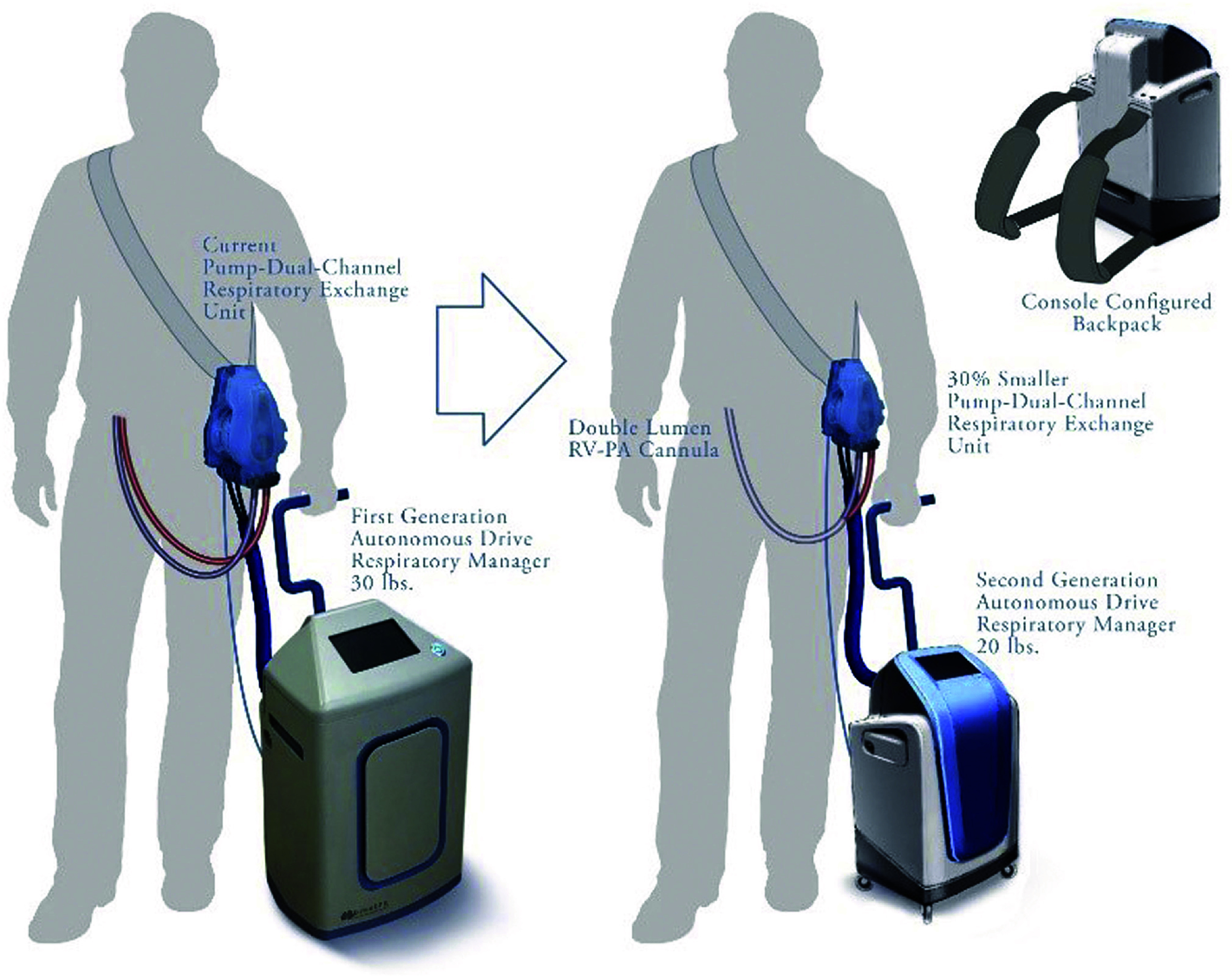
The Breethe OXY-1 system (Abiomed, Danvers, Mass., USA) included a wearable pump-lung unit and a small gas-free roller console. A lighter pumplung unit with a smaller console, possibly placed in a backpack, is under development. RV-PA, Right ventricle to pulmonary artery. (Adopted from Griffith et al., 2021 [145]. Copyright, 2021 The Authors. Published by Elsevier Inc. on behalf of the American Association for Thoracic Surgery. This is an open-access article under the CC BY-NC-ND license (http://creativecommons.org/licenses/by-nc-nd/4.0/).)

In 2014, Griffith, Wu, and Zhang began developing the Breethe OXY-1, a mobile, paracorporeal ECMO system intended for use outside the intensive-care setting, potentially in hospital wards or even at home [145]. Still, at the experimental stage, it connects to the patient through standard commercial cannulae. It can provide either venovenous or venoarterial support, depending on whether the return line is placed in a vein or an artery. A wearable pump-lung unit houses a centrifugal blood pump mounted on hybrid magnetic-hydrodynamic bearings and a purpose-built hollow-fibre oxygenator. These components are connected to a management console, resembling a wheeled carry-on luggage, which houses a battery unit and a fan that draws atmospheric air when compressed oxygen is unavailable (Figure 16) [145]. Although the concept is highly promising, substantial further refinement and clinical evaluation are required before its benefits can be confirmed. Should these hurdles be overcome, the Breethe OXY-1 may one day enable ward-level or even fully ambulatory ECMO.

These innovations highlight the expanding role of extracorporeal life support technology, which extends beyond mere life support to include the restoration of organ function and the improvement of patient recovery and quality of life.

## Summary

This article offers a concise yet comprehensive overview of the history and advancements in ECMO. However, this review has limitations, such as the expected length of a journal article and the potentially subjective selection of events and innovations. Nevertheless, the substantial amount of reviewed and referenced literature enhances the value of this paper.

**Figure 17:**
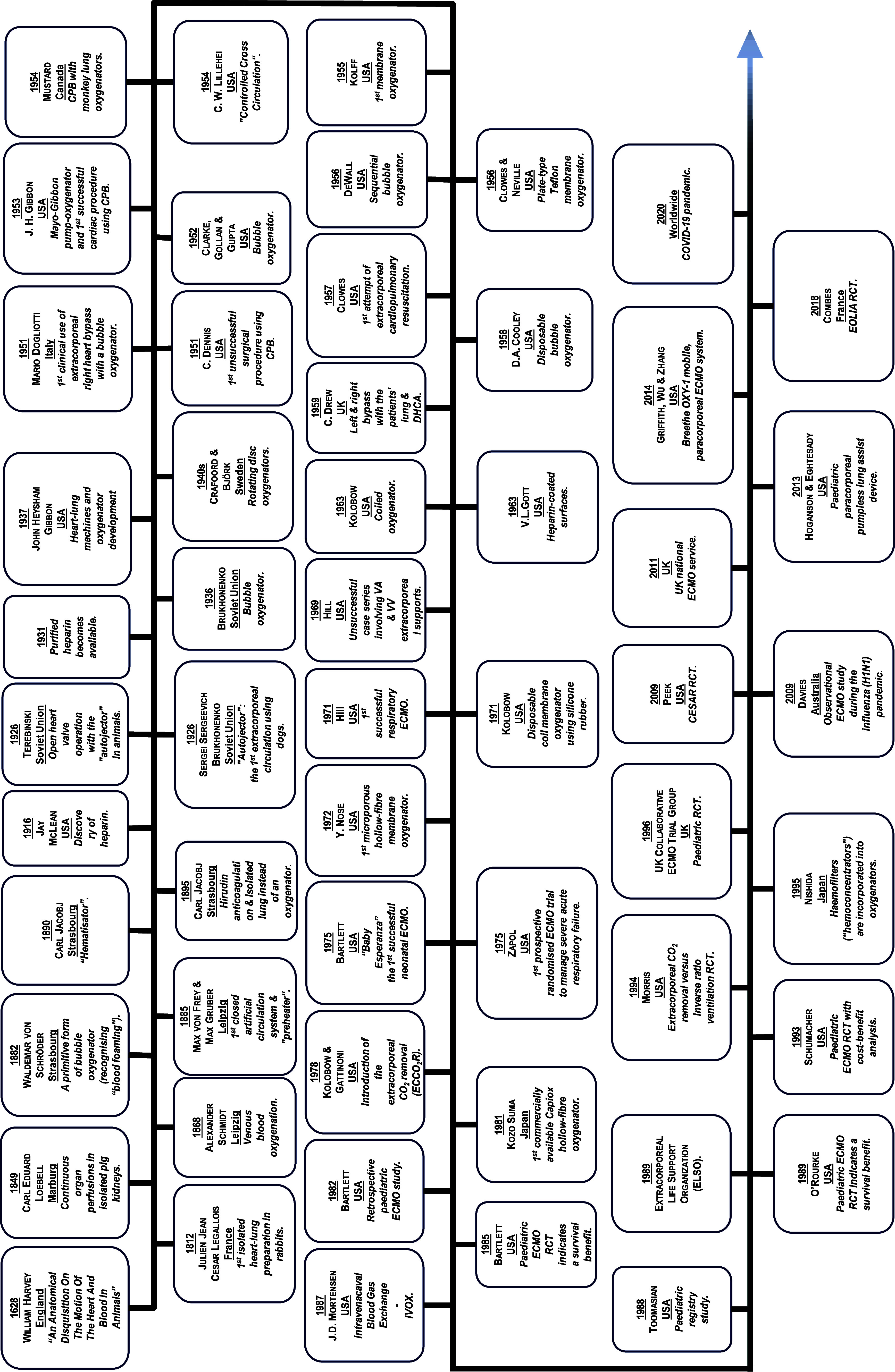
Timeline of ECMO. (USA, United States of America; ECMO, extracorporeal membrane oxygenation; CPB, cardiopulmonary bypass; VA, venoarterial; VV, venovenous; UK, United Kingdom; RCT, randomised controlled trial; COVID-19, coronavirus disease 2019.)

It took over 300 years from the circulatory system’s first description to initial oxygenator designs. The first clinical use of extracorporeal technology was in 1951 [9, 10], and Gibbon’s first successful cardiac procedure using CPB followed in 1953 [9, 10], establishing the role of CPB technology. CPB advancements paved the way for ECMO, leading to the first successful clinical case of VA-ECMO for respiratory failure in 1971 [1] and the first neonatal VA-ECMO in 1975 [22, 28, 29]. Initially, paediatric practice achieved the most success, but after the CESAR trial in 2009 [57], ECMO became globally established for adult and paediatric care. Figure 17 illustrates the timeline of ECMO.

Modern oxygenators and pumps minimise blood damage, enabling compact designs. Future innovations, such as improved surface coatings, endothelialisation and AI technology, push the boundaries and may position ECMO as a long-term support for further enhancing survival and quality of life [22]. This exponential progress in ECMO technology and clinical applications is a testament to the perseverance and ingenuity of early pioneers and recent innovators.

## Data Availability

No new data were generated or analysed in this study. All materials and sources are available in the public domain and are cited within the article’s reference list.
